# Impact of Anthropogenic Pollution on the Plant Species Diversity and Composition Along the Riparian Ecotones of Goa's Sal and Zuari Rivers

**DOI:** 10.1002/pei3.70037

**Published:** 2025-03-07

**Authors:** Moses Musisi, Celly Quadros, Krishnan Sellappan

**Affiliations:** ^1^ Department of Science, National Teachers' College, Kaliro Kyambogo University Kaliro Uganda; ^2^ Botany Discipline, School of Biological Sciences and Biotechnology Goa University Taleigao Goa India; ^3^ Department of Botany Government College of Arts and Sciences, Quepem Taleigao Goa India

**Keywords:** anthropogenic pollution, ecotone, hemeroby, plant species diversity, riparian plants, species composition

## Abstract

Anthropogenic pressures are increasingly constraining the health of riparian ecosystems by exposing their remnant vegetation to edge effects. Despite being at the land–water interface, conservation efforts have often overlooked how water pollution may indirectly exacerbate the broader impacts of anthropogenic pressures on riparian vegetation along riparian ecotones. This study therefore examined the impacts of anthropogenic pollution on plant species diversity and composition in riparian ecosystems. Transect and Quadrat methods were used to collect vegetation data. We also measured the physicochemical properties of the water samples. We used partial redundancy analysis (RDA), generalized linear models (GLMs), analysis of variance (ANOVA), and Tukey's HSD test for data analysis using R software version 4.3.2. The study identified 126 plant species from 45 families, with the Shannon–Wiener diversity index ranging from 2.06 to 3.10. Anthropogenic disturbances were generally at the alpha eu‐hemerobic level, characterized by strong human impacts. Redundancy analysis showed that the nature of human activities, hemeroby, and turbidity were the dominant explanatory factors affecting plant species composition. GLM regression revealed that anthropogenic disturbances (hemeroby) had a significant negative impact on riparian plant species diversity mediated by water pollution. The findings indicate that anthropogenic disturbances coupled with their detrimental effects on water quality lead to a decrease in plant species richness and the dominance of a select few plant species. This will ultimately lead to a decline in the overall plant species diversity. Our findings show that anthropogenic disturbances negatively impact plant species diversity and composition through altering the water quality and habitat degradation. The findings therefore highlight the critical need for stakeholders to prioritize sustainable practices that mitigate water pollution and reduce direct human disturbances. This will safeguard biodiversity and ecosystem functionality in riparian zones, thus ensuring the long‐term stability of environmental services that benefit both nature and human communities.

## 1 Introduction

Riparian plants are embedded within one of the most vulnerable and threatened ecosystems in the world today (Tockner and Stanford 2002). This riverine ecosystem is under stress due to extensive anthropogenic activities, mainly urbanization, agriculture, and industrialization, which have degraded its processes' integrity at both temporal and spatial scales (Yang et al. 2022). These human disturbances and pressures have both direct and indirect impacts on riparian plant species diversity and composition. As a result, plant communities along riparian ecotones are changing more swiftly and unpredictably, which reduces their ability as ecological engineers to restore the health of rivers (Abbas et al. 2021; Koskey et al. 2021).

Anthropogenic pollution refers to adverse alterations in the natural environment as a result of human activity (Arihilam and Arihilam 2019). It triggers enormous direct and indirect stressors that derail the riparian ecosystem's functioning, and in the process, riparian plants suffer immediate effects (Naiman et al. 2005; Hoppenreijs et al. 2022). This is attributed to the fact that anthropogenic pollution exposes the remnant vegetation to edge effects, a situation in which vegetation is exposed to environmental conditions of an entirely new ecosystem due to alterations in its original environmental resources (Koskey et al. 2021; Šálek et al. 2013; Ren et al. 2019). When these alterations dramatically modify the environmental conditions, changes occur in plant species diversity with the loss of local species of plants (Debinski and Holt 2000).

Direct effects, such as habitat destruction, land use changes, and physical modification of waterways, have immediate consequences on riparian ecosystems and are easily noticeable (Gu and Li 2024). These actions often lead to habitat fragmentation, loss of vegetation, and the disruption of ecological processes that support species richness (SR) and ecological health (de Magalhães et al. 2022). For example, cultivation leads to direct local destruction of vegetation and excessive siltation, which modify the fluvial geomorphology of the river channel (Jacks 2019). Additionally, excessive livestock grazing constrains plant vigor, which affects plant age structure and species diversity (Gatica et al. 2020). Such effects are easily visible and have been the focus of many interventions to restore and conserve riparian zones.

However, growing evidence suggests that anthropogenic activities may have profound and far‐reaching consequences on riparian species diversity and composition through indirect pathways (Zhang et al. 2023). Because riparian ecotones lie along the land–water interface, water quality may present an indirect pathway for anthropogenic effects on riparian vegetation (Sharma et al. 2021). The indirect effects through water pollution are often more subtle and difficult to detect than direct habitat loss, yet they can have long‐term and potentially irreversible impacts on biodiversity (Liu et al. 2015). Pollutants from agricultural runoff, industrial discharges, and urban wastewater introduce harmful substances into waterways, including excess nutrients, for example, nitrates and phosphates, heavy metals, and toxic chemicals (Smith and Siciliano 2015). These pollutants can alter water quality, leading to eutrophication, oxygen depletion, and the disruption of aquatic food webs, which in turn affects riparian species that depend on these water bodies for survival.

Water pollution exacerbates the decline in species diversity by altering the physical and chemical conditions of riparian habitats, which can lead to a shift in species composition, favoring pollution‐tolerant species while displacing sensitive species (Fares et al. 2020). This change can reduce the overall ecological integrity and resilience of riparian ecosystems. For example, urbanization, which the Sal and Zuari rivers are heavily exposed to, increases hard surface area, resulting in a decrease in soil permeability, lower groundwater tables, and high‐speed nutrient‐rich surface runoffs, while on the other hand, cultivation can lead to pesticide pollution (Koskey et al. 2021).

Various stakeholders have made efforts to improve the river quality of both the Sal and Zuari rivers (The Goan Network 2022, December 16). The Government of Goa's River Rejuvenation Project, launched in 2019 with an action plan to, among other things, rehabilitate the riparian zones, is the largest intervention to date [Goa State Pollution Control Board (GSPCB) 2019]. Although the extent of this intervention's success has not been thoroughly established or documented, the project has shown a keen interest in the connection between riparian vegetation and the preservation and improvement of riverine ecosystems.

It is against this background that this study was conceived to investigate the effects of anthropogenic disturbances and water pollution on plant diversity and composition in riparian ecosystems. It sought to identify how hemeroby and water quality independently and interactively influence plant community composition and diversity in the transitional ecotones between aquatic and terrestrial environments. We were therefore guided by the following research objectives: (1) To examine the variation in alpha and beta diversity of plant communities among the study stations and seasons, (2) to assess the levels of anthropogenic pollution along rivers, (3) to analyze the effect of anthropogenic pollution on the riparian plant species composition, and (4) to examine the effect of anthropogenic disturbances on the riparian plant species diversity and the mediating effect of water pollution. This study provides empirical evidence of how anthropogenic disturbances specifically alter plant species composition, degrade habitats, and affect water quality, leading to biodiversity loss. By identifying the cascading effects of these disturbances, this study offers new insights into the mechanisms driving biodiversity shifts. It also highlights the interconnected negative impact of habitat and water quality degradation on plant community structures, stressing the need for integrative conservation approaches.

## 2 Materials and Methods

### 2.1 Study Area

**FIGURE 1:**
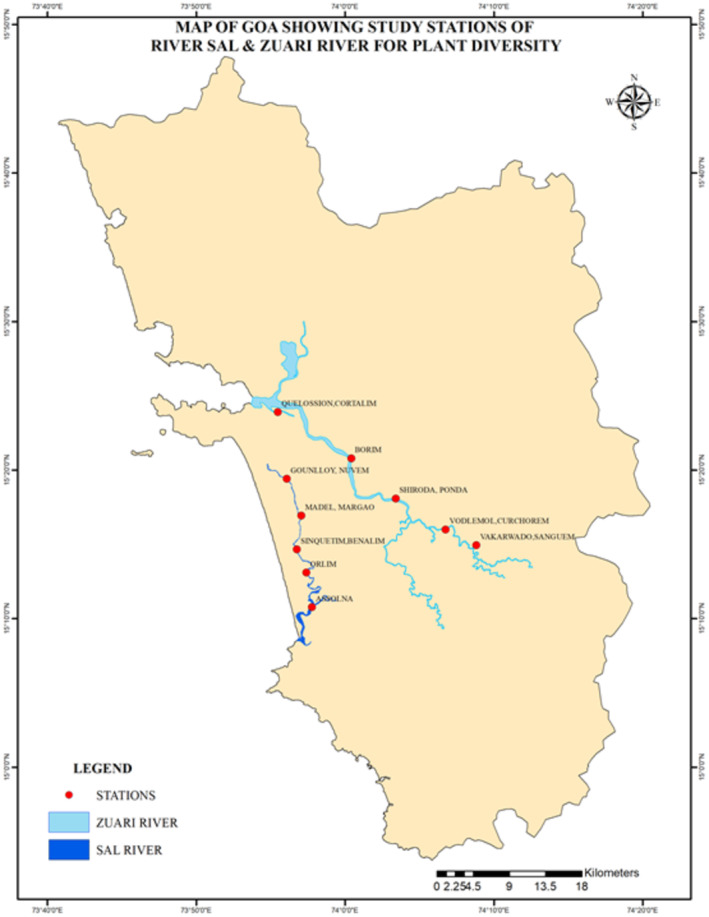
Map of Goa showing the study stations.

River Sal and River Zuari are key co‐influential rivers found in South Goa, India (Rodrigues 2022). South Goa is found between 15°44′30″—14°53′30″ N and 73°45′—74°26′ E, along the Indian Central Western Coast (Anant 2012). River Sal, dubbed the lifeline of Salcette, is a major source of water in the South Goa district, stretching about 40 km till it discharges into the Arabian Sea (Harmalkar 2023, January 19). Currently, the Sal River is ranked the most polluted river in Goa by the Central Pollution Control Board, with a stretch of about 22 km considered so polluted that it is unsuitable for bathing, fishing, or other recreational activities (GSPCB 2019). Mankind's interference is very evident along the river. Over the years, the river has become an ecological catastrophe due to an expansion in urbanization, drastic land use, careless hill cutting, encroachments, rubbish dumping, and frequent human meddling (Nandkumar 2009, April 6) (Figure 1).

Just like the Sal River, the Zuari River is facing tremendous ecological pressure from anthropogenic activities and hence likewise needs eco‐restoration [River Rejuvenation Committee (RRC) 2019]. It is the longest river in Goa, stretching about 145 km till it also discharges into the Arabian Sea (RRC, 2019). The Central Pollution Control Board has classified it under priority V, deeming certain portions unsafe for bathing and recreational services (GSPCB 2019).

The study area is located in the Torrid Zone and has a tropical monsoon climate that is characterized by being hot and humid for most of the year [Department of Information and Publicity Goa (DIPG) 2023]. The maximum temperatures in this area range between 28°C and 33°C, while minimum temperatures range from 20°C to 26°C (DIPG 2023). On average, May is the hottest month with high humidity and temperatures that can rise beyond 35°C, while January is the coldest month with temperatures that can fall as low as 19°C (Indian Meteorological Department 2023). The seasonal distribution of precipitation is not uniform; more than 95% of that rainfall falls during the monsoon season (June to October), with July being the wettest, receiving an average of 995 mm of rain (DIPG 2023; Indian Meteorological Department 2023).

### 2.2 Data Collection

We divided each river into five segments or strata of equal length along its course. We randomly selected one sampling station within each stratum, resulting in five sampling stations per river and 10 sampling stations altogether. We conducted an exploratory survey to identify the various plant communities at the study stations. The plant survey was conducted thrice, once in every season, that is, post‐monsoon, monsoon, and pre‐monsoon. At each station, a line transect 50 m long was established perpendicular to the river. We sampled trees that fell within 5 m on either side of the transect line. Every 10 m along the transect line, we established quadrats with plots measuring 5 m by 5 m on alternating sides. In these plots, we sampled shrubs and saplings, while within the same plots; we used subplots of 1 m by 1 m for grasses. Given the sparse distribution of trees in the study area, using a combination of the line transect and quadrat methods ensured that samples were representative of all the different layers of vegetation. We combined all identified plants along the transect line, subplots, and in the full plot to form a composite sample of the study station. We listed the plants and estimated their abundance. These plants were identified to the species level, and voucher specimens were collected. We collected and later identified plants that were unidentifiable in the field using their voucher specimens, referencing herbaria and expert assessment.

We collected water in bottles, filled them as full as possible without leaving any air inside, and sealed them tightly. We labeled them with the collection date, time, and study site before keeping them in a dark box. We immediately recorded the dissolved oxygen, pH, and temperature of the water. We used the PCTestr 35 multi‐parameter analyzer conductivity meter to measure electrical conductance (EC) and the AED08 Dissolved Oxygen Kit to measure BOD. We used a thermometer to measure temperature and a Secchi disc to measure turbidity. Bicarbonate (HCO_3_^−^) ions were estimated by titration with sulfuric acid (APHA 2012), NO_3_^−^, and PO_4_^3−^by a spectrophotometric method (Doane and Horwáth 2003) and Ca^2+^ and Mg^2+^ by EDTA titration with a murexide indicator (Chang 2010).

### 2.3 Water Pollution Index

We computed the water pollution index (WPI) following the formula below by Horton (1965) as modified by Hossain and Patra (2020).

$$\mathrm{WPI}=1/n\sum_{i=1}^{n}\mathrm{PLi}$$

where *n* is the number of parameters and PLi is the pollution load of *i*th parameter. The pollution load (PLi) was calculated beforehand using the following formula:

$$\mathrm{PLi}=1+\frac{\left(\mathrm{Ci}-\mathrm{Si}\right)}{\mathrm{Si}}$$

where Ci is the observed concentration of i th parameter, Si is the standard or highest permissible limit for the respective parameter.

### 2.4 Hemeroby (Anthropogenic Disturbance) Index

We determined the anthropogenic disturbance of the study area using the Hemeroby Index by Blume and Sukopp (1976). This index is an indicator of the extent at which the vegetation of an area has drifted from its natural state due to anthropogenic activities. It has a scale of 1 to 7 (Table 1). The hemoroby index is calculated using the formula by Steinhardt et al. (1999) given as:

$$M=100/n\sum_{h=1}^{n}\mathrm{fnh}$$

**TABLE 1:** Degrees of hemeroby.

Hemeroby level	Description	Anthropogenic pressure intensity	Hemeroby factor
A‐hemerobic	Almost no human impacts Natural	Lack of anthropogenic impact, flora and vegetation unaffected by human pressure. Bare rocks	1
Oligo‐hemerobic	Weak human impacts Close to natural	Minor anthropogenic impacts are observed; however, they do not modify the substrate. Broad‐leaved forest, coniferous forest, mixed forest, beaches, dunes, sands	2
Meso‐hemerobic	Moderate human impacts Semi‐natural	Weak to moderate, or periodic anthropogenic factors. Transitional woodland‐shrub, mixed forest (not PNV), Sparsely vegetated areas, natural grasslands, burnt areas, moors and heathland	3
Beta Eu‐hemerobic	Moderate to strong human impacts Relatively far from natural	Continuous and strong anthropogenic impacts causing strong modifications of the substrate. Green urban areas, water courses, pastures, land principally occupied by agriculture, with significant areas of natural vegetation	4
Alpha Eu‐hemerobic	Strong human impacts Far from natural	Sport and leisure facilities, non‐irrigated arable land, vineyards, complex cultivation patterns, fruit trees and berry plantations	5
Poly‐hemerobic	Very strong human impacts Strange to natural	Continuous and very strong anthropogenic impacts. Vegetation is characterized by a high degree of specialization and pioneer nature. Discontinuous urban fabric, construction sites, mineral extraction sites, dump sites	6
Meta‐hemerobic	Excessively strong human impacts Artificial	Continuous impact of anthropogenic factors that are so strong they exceed the tolerance of plants. Continuous urban fabric, port areas, industrial or commercial units, airports biocoenosis destroyed, road and rail networks and associated land	7

*Source:* Adopted from Blume and Sukopp (1976) and Rüdisser et al. (2012).

where *M* is the hemeroby index, *n* is the number of degrees of hemeroby, *fn* is the proportion of the category *n*, and *h* is the hemeroby factor.

### 2.5 Alpha Diversity

Riparian plant species diversity was determined by the Shannon–Wiener index of diversity (1949), calculated using the equation below;

$$H^{\prime }=-\sum_{i}^{n}\mathrm{pi}.\ln.\mathrm{pi}$$

Where $H^{\prime }$ is Shannon–Wiener diversity index and $\;p^{i}$ represents is the proportion of the total number of all species in a plot and *In* is natural logarithm. We used the Pielou Index (1977) to indicate homogeneity and heterogeneity (evenness) of plant species at a study site;

$$\mathrm{Jsw}=\frac{H^{\prime }}{\text{In}S}$$

Where *H*′ is the Shannon–Wiener diversity index and S is the total number of species at a site. We used Margalef's richness index and the formula to indicate the species richness;

$$\;\text{Species richness}=\frac{S-1}{\text{In}\;N.}$$

where *S* is the number of species and *N* is the total number of individuals.

### 2.6 Statistical Analysis

The data was transformed prior to analysis. The species abundance data had many zeros, hence it was reliable to the double zero problem. This data was therefore subjected to the Hellinger transformation performed using the “decostand ()” function from the vegan package. This transformation expresses the species' abundances as the square root of their relative abundance at each study station (Borcard, Gillet, and Legendre 2011). Due to the high sensitivity of the constrained ordination methods to collinearities in the explanatory matrix, we checked for correlations among the explanatory variables, and for variables with *r* > 0.7, one was excluded. We excluded bicarbonate and nitrate ion concentrations in this process because of their strong collinearity with pH and hemeroby, respectively. The latter were retained at the expense of the former through the ecological reasoning technique. Second, we applied the variance inflation factor (VIF) to the variables selected by the first correlation, after calculating the global partial redundancy analysis (RDA) using the “vif.cca()” function. We used the “forward.sel()” function to select the best environmental variable for the study. pH, biochemical oxygen demand (BOD), and water hardness were found to be redundant and thus eliminated with the second criteria. The environmental data was standardized using the “decostand ()” function because the variables had different units. We used the Bray–Curtis dissimilarity index to analyze beta diversity and compare plant communities among the study stations and rivers using the “vegdist” function.

To investigate the influence of anthropogenic pollution variables (explanatory variables) on the species composition (response variables), we used RDA. This analysis enables the display of the patterns of the response data uniquely explained by a linear model of the explanatory variables when the effect of other covariates is held constant (Borcard, Gillet, and Legendre 2011). As such, in this study, the effect of anthropogenic pollution on species composition was assessed while taking into account the variation due to salinity, which was not a focus of this study. This was because the variation in salinity was largely caused by the mixing of seawater with river water, hence not mainly due to anthropogenic activities, which were the study's focus. The RDA was performed using the “rda ()” function from the vegan package, after which the statistical significance was tested using the “anova.cca” function. Before analysis, we ensured the adequacy of the data by checking for normality and homogeneity of variance after transformation.

To analyze the impact of anthropogenic pollution variables on plant species diversity, richness, and evenness, we employed general linear models (GLMs). We used the water physicochemical variables to determine the WPI. Hemeroby was used as the explanatory variable, the WPI as the mediating variable, and the SR and richness indices as the response variables. The family distribution for the response variables was checked; as such, SR and diversity were run with the Gaussian model with the “log” link. Three parameter models were used in the analysis: model 1 regressed the explanatory variable with the response variables, model 2 regressed the response variables simultaneously with the explanatory and mediating variable, while model 3 regressed the mediating variable with the response variables. We used the Akaike information criterion (AIC) model selection technique to select the best model among the tested models for each response variable using the “AICcmodavg” package. We used the “aov()” function to find out how the number, evenness, and diversity of plant species varied between study sites and between seasons. We then used the “Tukey HSD” function to see how different sites or seasons were from each other. We performed all analyses using R software version 4.3.2.

## 3 Results

### 3.1 Riparian Plant Species Alpha Diversity

The study recorded a total of 126 plant species belonging to 106 genera and 45 families (Appendix A). Specifically, Fabaceae (16 species), Cyperaceae (13 species), Poaceae (13 species), Asteraceae (7 species), and Lamiaceae (6 species) were the most species‐rich families in the study area. Other families with a significant representation of species included Acanthaceae, Araceae, Moraceae, and Convolvulaceae, with four species each, and Vitaceae, Polygonaceae, Amaranthaceae, Malvaceae, and Rhizophoraceae, with three species each. The study findings also revealed that about half of the families were represented by a single species and six families by two species.

The study results revealed that the plant SR varied between a Margalef's index of 7.076 (very high) and 2.926 (very low). Meanwhile, the Shannon–Wiener species diversity index ranged between 3.10 (very high) and 2.06 (moderately low). On the other hand, the Pielou's species evenness index was generally high among all study sites with an average of 0.822 and a minimum of 0.733 (Table 2).

**TABLE 2:** Characteristics of the study sites.

Station	SR	SD	SE	HEM	WPI	N0_3_‐(ppm)	PO_4_^3−^(ppm)	pH	Turb (FNU)	Temp (°C)	EC (mS)
Z1A	5.835	2.83	0.824	3.0	0.312	2.90	0.18	7.05	0.018	28.0	0.58
Z1B	5.003	2.41	0.733	3.5	0.375	3.10	0.11	7.07	0.028	28.5	0.91
Z1C	4.542	2.28	0.718	3.5	0.407	3.50	0.25	7.13	0.027	28.5	6.40
Z2A	5.705	2.73	0.828	4.5	0.546	10.8	1.15	7.04	0.030	30.0	0.70
Z2B	5.815	2.29	0.703	4.7	0.663	11.3	1.31	7.05	0.030	30.0	0.86
Z2C	5.683	2.22	0.700	4.8	0.65	11.2	1.29	6.97	0.035	30.5	0.79
Z3A	7.794	3.32	0.893	3.9	0.395	3.80	0.55	6.92	0.077	29.0	5.91
Z3B	7.466	3.22	0.878	4.0	0.421	3.60	0.92	6.98	0.145	28.0	7.35
Z3C	5.968	2.72	0.813	4.1	0.448	4.00	1.38	6.82	0.086	29.0	13.99
Z4A	5.448	2.96	0.878	4.1	0.425	3.40	0.63	7.06	0.079	29.0	10.5
Z4B	5.500	2.85	0.855	4.0	0.432	3.20	0.91	7.08	0.116	29.0	14.6
Z4C	4.423	2.69	0.869	4.0	0.495	3.90	0.65	7.04	0.081	29.5	20.0
Z5A	6.015	2.51	0.825	4.5	0.491	7.40	0.41	7.55	0.145	29.5	22.0
Z5B	6.431	2.48	0.875	4.9	0.496	8.00	0.38	7.65	0.134	30.0	24.5
Z5C	5.625	2.03	0.815	5.0	0.531	7.70	0.14	7.32	0.124	29.5	29.3
S1A	3.917	2.53	0.807	3.1	0.372	8.30	0.64	7.12	0.051	25.0	1.09
S1B	3.856	2.57	0.833	4.0	0.416	9.00	0.50	7.18	0.045	27.0	2.33
S1C	3.551	2.38	0.794	3.8	0.488	9.20	0.88	7.56	0.055	25.0	3.40
S2A	3.924	2.53	0.806	4.9	0.684	12.1	0.91	6.92	0.211	31.0	0.24
S2B	3.899	2.37	0.767	5.2	0.89	11.9	1.10	6.81	0.299	30.5	0.88
S2C	2.909	2.17	0.783	5.8	0.97	12.8	1.20	6.4	0.398	30.5	0.98
S3A	3.730	2.72	0.894	4.4	0.575	11.7	0.70	7.01	0.084	30.0	2.30
S3B	3.916	2.67	0.876	4.9	0.636	12.5	0.77	6.91	0.081	30.0	3.40
S3C	3.053	2.26	0.816	5.0	0.699	13.0	1.23	6.7	0.095	30.5	5.20
S4A	5.876	3.07	0.887	3.5	0.341	3.30	0.41	7.21	0.030	26.0	9.12
S4B	5.780	2.99	0.88	3.8	0.418	2.50	0.54	7.15	0.035	26.5	11.27
S4C	4.557	2.54	0.799	3.8	0.458	3.20	0.55	6.89	0.037	27.0	12.99
S5A	3.710	2.45	0.865	4.9	0.612	6.50	0.44	7.81	0.051	30.0	20.0
S5B	3.119	2.2	0.856	5.9	0.703	7.40	0.51	7.73	0.053	30.0	22.5
S5C	1.949	1.52	0.784	6.0	0.706	7.30	0.93	7.15	0.060	29.0	25.1

Abbreviations: A, Monsoon; B, Post‐monsoon; C, Pre‐monsoon; EC, Electrical conductivity; HEM, Hemeroby; NO_3_, Nitrate; PO_4_, Phosphate; S, River Sal; SD, Species diversity; SE, Species evenness; SR, Species richness; Temp, Temperature; WPI, Water pollution Index; Z, River Zuari.

River Zuari had a higher Shannon–Wiener species diversity index (M = 2.636 ± 0.368) as compared to that of River Sal (M = 2.465 ± 0.367). Field data showed that River Zuari possessed 87 different plant species as compared to 68 species found along the Sal River. Tree species belonging to the family Fabaceae, including *Acacia chundra* and *Pongamia pinnata* , along with those from the family Moraceae, including *Artocarpus heterophyllus, Ficus hispida, and Ficus recemosa*, were unique to the Zuari River. Species unique to River Sal were mainly herbs or shrubs, for example, *Brachiaria mutica* , *Ipomoea pes‐caprae*, and *Crotalaria pallida* .

#### 3.1.1 Variation in Alpha Diversity Across the Study Seasons and Stations

**FIGURE 2:**
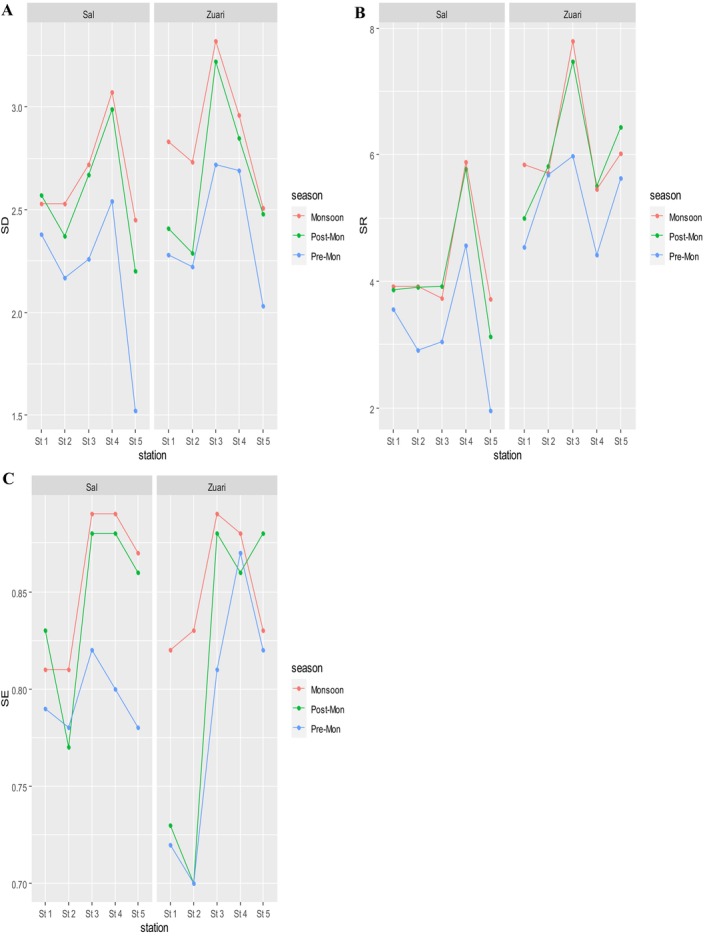
Variable plots showing the variation of (A) plant species diversity (SD), (B) plant species richness (SR), and (C) Plant species evenness (SE) along the Sal and Zuari rivers across the study seasons (Po‐Mon: Post‐monsoon, Pre‐Mon: Pre‐monsoon).

The Shannon–Wiener plant species diversity decreased across the seasons from the Monsoon through the post‐monsoon season to the pre‐monsoon season (Figure 2A). This implies that the plant species became less even, rich, and diverse as the weather conditions became drier. Specifically, the study field findings showed that many annual herbs were absent in either the post‐monsoon or pre‐monsoon period. Species such as *Chloris barbata* and *Ehrharta erecta* (grasses), along with others like *Cyperus javanicus* , *Ipomoea corymbosa* , *Ipomoea pes‐caprae* , and *Persicaria maculosa* , were notably absent in the post‐ or pre‐monsoon period.

Analysis of variance (ANOVA) results revealed that there was a significant difference in species diversity (F (2,27) = 5.904, *p* = 0.007) and evenness (F(2,27) = 3.557, *p* = 0.043) across the study seasons but no significant difference in SR (F (2,27) = 1.538, *p* = 0.233). This implies that across the seasons, the number of species does not change considerably (Figure 2B), but their evenness (Figure 2C) and diversity are altered significantly. This means that the abundance of individuals of some plant species decreased as that of other species increased or remained almost constant. For example, in the post‐ and pre‐monsoon seasons, the abundance of most herbaceous plants declined. However, in the same period, the species primarily from the Asteraceae family, such as *Chromolaena odorata, Sphagneticola trilobata*, and *Tridax procumbens* , along with other species like *Phyllanthus reticulatus, Rhizophora mucronata*, and *Pontederia crassipes*, increased in abundance instead. When multiple comparisons were made between individual seasons using the Tukey's HSD test, it was found out that the mean species diversity and species evenness were not significantly different between successive seasons, i.e., monsoon to post‐Monsoon (*p* = 0.0513 and *p* = 0.0534) or post‐monsoon to pre‐monsoon (*p* = 0.513 and *p* = 0.275), but it was instead significantly different when considered across the entire seasons from monsoon to pre‐Monsoon (*p* = 0.006, 95% CI = [−0.1958–0.5158] and *p* = 0.034, 95% CI = [0.004–0.192]). These results imply that across seasons, plant species diversity does not drastically change; instead, there is a gradual variation from one season to another, and by the end of the three seasons, differences become much more pronounced.

Among the study stations, study results show that the Shannon–Wiener plant species diversity generally increased downstream but decreased toward the rivers' estuaries (Figure 2A). Specifically, the diversity indices were highest at the third and fourth study stations. Field data showed that a number of halophyte species started to appear at the third stations, which were midway along the rivers, which may explain the increase in diversity. These halophytes were mainly the mangroves, their associates, and members of the family Cyperaceae. They included *Bruguiera gymnorrhiza, Rhizophora mucronata, Acanthus illicifolius*, and *Avicennia officinalis*, plus their associates like *Ipomoea violacea, Dolichandrone spathacea*, and *Derris trifoliata*. Although many mangroves continued to appear beyond station 4, it was observed that members of family Cyperaceae, including *Cyperus articulatus, Cyperus javanicus, Cyperus longus, Cyperus rotundus, Fimbristylis dichotoma*, and *Schoenoplectus lacustris*, did not exceed station 4. They were absent at the fifth stations, which were highly saline, hence a decrease in SR. Stations 3 and 4 had a large number of species, which were also highly abundant. These were mostly members of the families Poaceae, Cyperaceae, Acanthaceae, and Rhizophoraceae, hence the high diversity at these stations. The upper study stations 1 and 2 also had a large species especially from families Asteraceae and Fabaceae. However, a majority of these species were less abundant making these study stations less diverse compared to 3 and 4. On the other hand, the study findings showed that sites that were far downstream, specifically, the fifth stations, had the least number of species. These were mainly from families Acanthaceae and Rhizophoraceae but were also significantly less abundant.

#### 3.1.2 Variation in Beta Diversity Among the Study Stations

**FIGURE 3:**
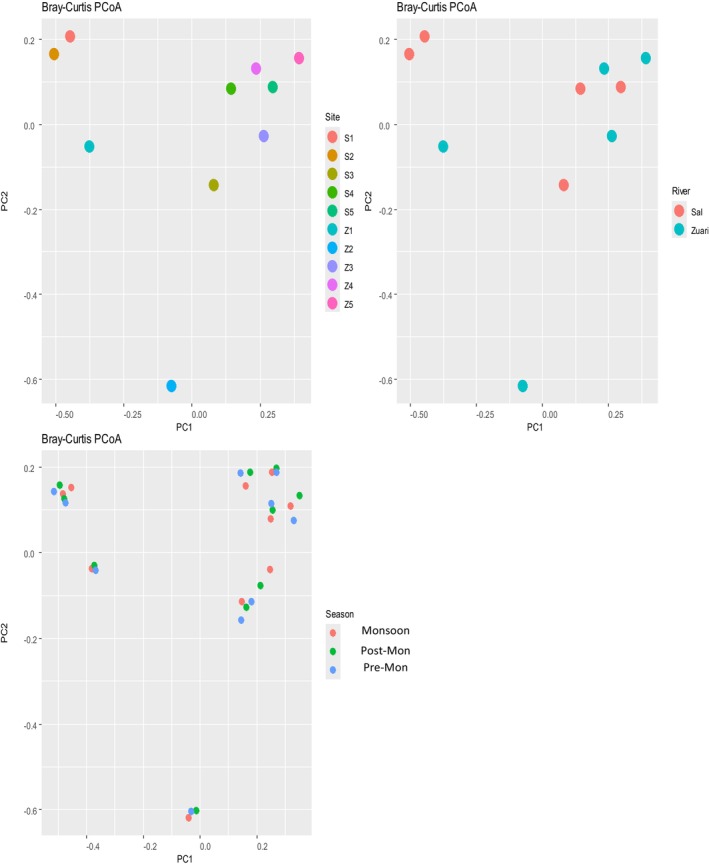
Beta diversity plots by A‐Study sites, B‐Rivers, and C‐Seasons.

We used the Bray–Curtis dissimilarity index to compare plant communities among the study stations, rivers, and seasons (Table 3). The study results revealed that, generally, study stations further apart geographically along the river show greater dissimilarity, which indicates heterogeneity within the river (Figure 3A). As such, the dissimilarity between the first and last stations, that is, Z1 and Z5 respectively was 0.981, indicating a very high dissimilarity in plant communities at these two stations. Station Z2 generally showed the highest dissimilarity indices, suggesting that it had the most distinct plant community composition compared to other stations. On the other hand, stations closer to one another had low dissimilarity values; as such, station Z3 and Z4 had a dissimilarity index of 0.489, S4 and S5 had 0.597, and S1 and S2 had 0.566, which indicates moderate similarity in their plant compositions.

**TABLE 3:** The Bray–Curtis dissimilarity indices among the study stations.

SITES	Z1	Z2	Z3	Z4	Z5	S1	S2	S3	S4
Z2	0.876								
Z3	0.863	0.818							
Z4	0.849	0.888	**0.489**						
Z5	0.981	0.955	**0.589**	**0.502**					
S1	**0.645**	0.958	0.859	0.858	0.934				
S2	0.797	0.954	0.973	0.893	1	**0.566**			
S3	0.856	0.866	0.752	0.846	0.78	0.885	0.927		
S4	0.775	0.904	**0.634**	**0.633**	0.71	0.782	0.901	**0.613**	
S5	0.950	0.925	0.734	0.734	**0.623**	0.931	0.977	0.796	**0.597**

Abbreviations: 1–5, study stations; bold values, Moderate and low dissimilarity values; S, River Sal; Z, River Zuari.

Despite the overall differences between plant communities in Rivers Zuari and Sal, the Bray–Curtis dissimilarity index indicates that these differences were smaller when comparing stations with similar positions along each river (Figure 3 B). For example, the dissimilarity between Z4 and S4 is 0.633, while that between Z1 and S1 is 0.645. These are notably lower than the dissimilarities between stations further apart between the rivers, such as Z5 and S2, which is 1.00 (completely distinct communities). This suggests that plant community composition along the two rivers exhibits some parallels or shared environmental influences based on station position. Findings also show that across the seasons, there are slight changes in plant compositions at the study sites (Figure 3 C). Despite the changes being slight, results show that generally, the plant composition during the pre‐monsoon season is more dissimilar in comparison to other seasons.

### 3.2 Levels of Anthropogenic Pollution Along the Rivers

Results revealed that rivers were facing moderate human impacts to very strong human impacts, as shown by hemeroby levels ranging from 3.0 to 6.0. This implies that no station along both rivers was free of human disturbance. Specifically, the second and fifth stations faced the strongest human alterations at Alpha eu‐hemerobic or poly‐hemerobic levels, while the first stations were at the meso‐hemerobic disturbance level characterized by periodic human interference and generally spared of adverse human alterations. The hemerobic levels were generally higher along the Sal River as compared to the Zuari River. The major anthropogenic activities along the river included urbanization, fishing, sewage disposal, waste dumping (especially plastics), and agriculture. The river ecotone featured forested areas where human impact was minimal.

### 3.3 RDA Ordination and Distribution for Effect of Anthropogenic Pollution on Plant Species Composition

RDA results show that the selected anthropogenic pollution variables (Hemeroby, temperature, turbidity, and human activity) explained 54.8% of the variation in plant community composition across the study sites (Table 4). The controlled variable, salinity, explained 16.6% of the variation, while the residual variance was only the unconstrained 21.6%. This implies that the selected variables explained most of the variation in species composition. The results further show that our full model significantly explains 44.9% of the variation in plant species abundance across the study sites (*p* = 0.001). Every explanatory variable included in the analysis had a significant impact on the species composition (Table 5), but the strongest impact was shown by human activities (*R* ^2^ = 0.152), turbidity (*R* ^2^ = 0.079), and hemeroby (*R* ^2^ = 0.077). Phosphate ion concentration had the least significant impact of all environmental variables (*R* ^2^ = 0.049).

**TABLE 4:** RDA results.

	Inertia proportion		*F*	Pr(> *F*)	*R* ^2^	Adj *R* ^2^
Total	0.756	1.000	4.801	0.001***	0.548	0.449
Conditioned	0.126	0.166				
Constrained	0.415	0.548				
Unconstrained	0.216	0.286				

*** *p* < 0.001.

**TABLE 5:** Relationships between species composition and explanatory variables based on RDA.

Explanatory variable	*R* ^2^	*F*	*P*
Hemeroby	0.077	7.128	0.001***
Temperature	0.053	4.866	0.001***
Turbidity	0.079	7.297	0.001***
Phosphate	0.049	4.553	0.001***
Human activity	0.157	3.641	0.001***

*** *p* < 0.001.

**FIGURE 4:**
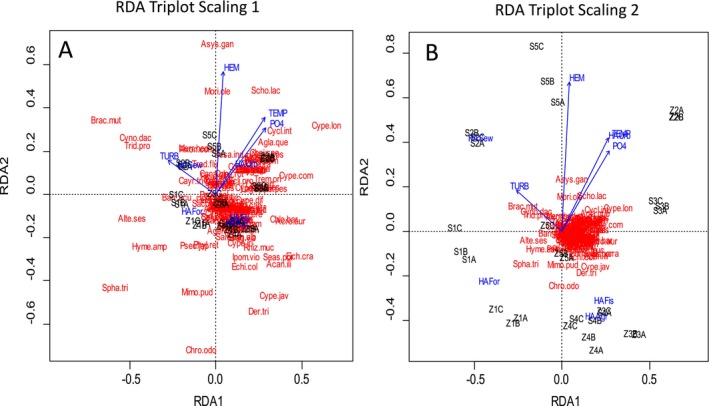
Redundancy analyses (RDA) plots of species composition and the environmental variables for both rivers. A‐scaling 1, B‐scaling 2. The arrows indicate how the variables are related to the RDA axes. Agr, agriculture; Fis, fishing; For, forestry; HA, human Activity; HEM, Hemeroby; PO_4_, phosphates; Sew, sewage; TEMP, temeprature; TURB, turbidity; Urb, urbanization. Acan.ill = Acanthus ilicifolius, Acme.rad = Acmella radicans, Acro.aur = Acrostichum aureum, Age.con = Ageratum conyzoides, Alt.ses = Alternanthera sessilis, Asys.gan = Asystasia gangetica, Avic.off = Avicennia officinalis, Axon.com = Axonopus compressus, Bar.acu = Barringtona acutangula, Bra.mut = Brachiaria mutica, Bru.gym = Bruguiera gymnorhiza, Cayr.tri = Cayratia trifolia, Chlo.bar = Chloris barbata, Chr.odo = Chromolaena odorata, Cro.ver = Crotaloria verrucosa, Cyno.dac = Cynodon dactylon, Cype.jav = Cyperus javanicus, Cype.lon = Cyperus longus, Cype.rot = Cyperus rotundus, Der.tri = Derris trifoliata, Pont.cra = Pontendiales crassipes, Hyme.amp = Hymenachne amplexicaulis, Ipom.pes = Ipomoea pes‐caprae, Ipom.vio = Ipomoea violacea, Mimo.pud = Mimosa pudica, Mori.ole = Moringa oleifera, Pand.tec = Pandans tectorius, Phyl.ret = Phyllanthus reticulatus, Rhiz.muc = Rhizophora mucronata, Scho.lac = Schoenoplectus lacustris, Seas.por = Seasuvium portulacastrum, Spha.tri= Sphagneticola trilobata, Trid.pro = Tridax procumbens.

The first axis of the RDA ordination plot (Figure 4B) was positively associated with hemeroby, temperature, phosphate, and human activities (urbanization, agriculture, and fishing) but negatively associated with turbidity and human activities (sewage disposal and forestry). Of the human activities, sewage disposal, urbanization, and forestry had the greatest impact on plant species abundance, while fishing had the least. As shown in Figure 4A, a large number of grasses (family Poaceae) including *Brachiaria mutica* , *Axonopus compressus,* and *Cynodon dactylon* were positively associated with sewage disposal, herbs including *Sphagneticola trilobata* , *Chromolaena odorata* , Acmella radicans, *Mimosa pudica* , *Hymenachne amplexicaulis* , and *Alternanthera sessilis* associated with forested areas that had less human alterations, trees including Avicennia officinalis, Barringtonia acutangula, and *Ipomoea pes‐caprae* mainly associated with urbanization, while the most mangroves and their associates including *Derris trifoliata* , *Cyperus javanicus* , *Ipomoea violacea,* and *Rhizophora mucronata* were positively associated with fishing activity. The majority of the mangrove species showed a positive correlation with the first axis, while the majority of the grasses showed a negative correlation with the same axis.

### 3.4 Effect of Anthropogenic Pollution on Plant Species Diversity

The effect of anthropogenic disturbances (Hemeroby) on riparian plant species diversity (Alpha diversity metrics) and the mediation effect of water pollution levels was analyzed using GLM regression analysis (Table 6).

**TABLE 6:** Guassian general linear model results.

Parameter model			Estimate	SE	T	Sigificance	AICc	AICcWt
SR	Model 1	HEM	−0.448	0.169	−2.65	0.001	84.33	0.05
	Model 2	HEM	0.187	0.559	0.569	0.574	81.48	0.23
		WPI	−0.733	0.314	−2.30	0.029		
	Model 3	WPI	−0.571	0.155	−3.66	0.001	79.19	0.72
SD	Model 1	HEM	−0.300	0.071	−4.22	0.000	76.29	0.61
	Model 2	HEM	.‐0.474	0.144	−1.58	0.124	78.60	0.19
		WPI	−0.172	0.699	−0.580	0.566		
	Model 3	WPI	−583	0.154	−3.79	0.001	78.60	0.19
SE	Model 1	HEM	−0.091	0.188	−0.548	0.588	90.79	. 0.13
	Model 2	HEM	0.605	0.350	1.698	0.101	88.12	0.49
		WPI	−0.803	0.350	−2.30	0.029		
	Model 3	WPI	−0.280	0.184	−1.61	0.119	88.59	0.39

Abbreviations: HEM, Hemeroby; SD, Species diversity; SE, Species evenness; SE, Standard error; SR, Species richness; WPI, Water pollution.

**FIGURE 5:**
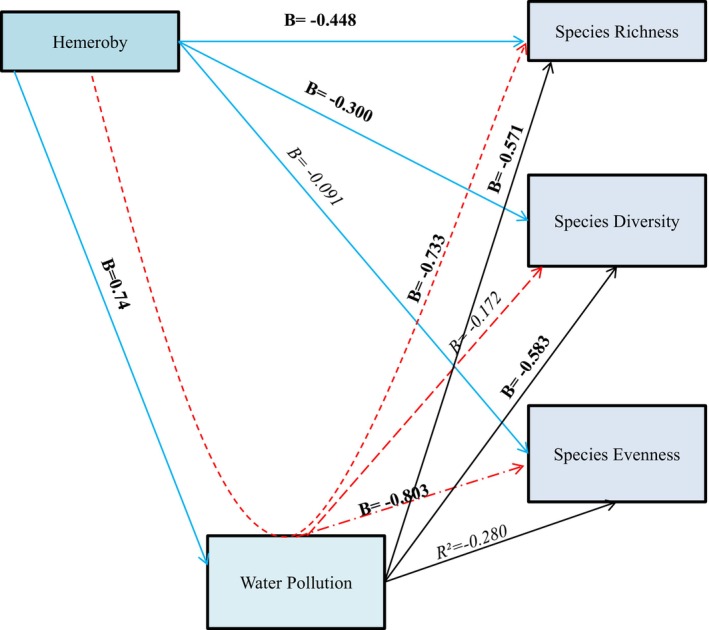
Results for the GLMs for the effect of anthropogenic pollution on plant species diversity, richness, and evenness. Blue lines‐Model 1, Red lines‐Model 2, Black lines‐Model 3.

**FIGURE 6:**
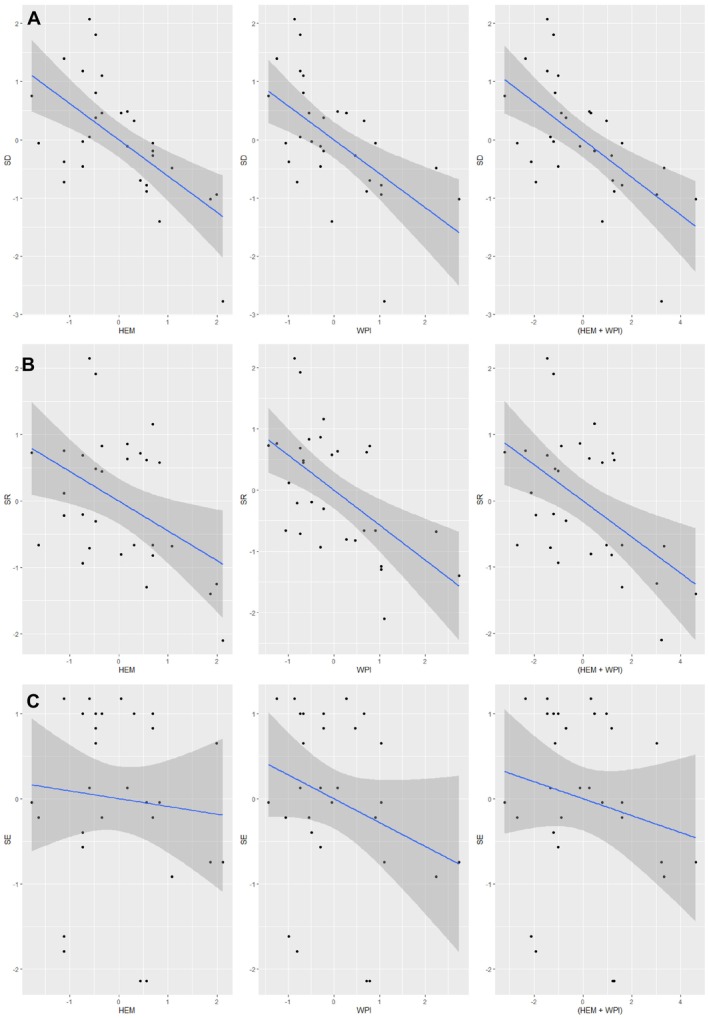
Parameter models showing the relationships between the hemeroby (HEM) (predictor variable), water pollution (WPI) (mediator variable), and response variables [species richness (SR), diversity (SD), and evenness (SE)]. The blue line represents the best fit of the generalized linear models to the data, and the gray shading area indicates the 95% confidence interval. The relationship among hemeroby, water pollution, and (A) species diversity, (B) species richness, and (C) species evenness.

For SR under models 1 and 3, the direct effects of hemeroby and water pollution on SR were both significant and negative (Model 1: B = −0.448, *p* = 0.001; Model 3: B = −0.571, *p* = 0.001). These findings indicate that increased human disturbance and reduced water quality independently lead to declines in plant SR. However, in Model 2, which examined the combined effects of hemeroby and water pollution, the influence of hemeroby became insignificant while water pollution remained significant (Figure 5). This suggests that water pollution mediates the relationship between hemeroby and SR, serving as a significant pathway through which human disturbances impact plant diversity. AIC model selection criteria further supported this conclusion. Model 3, which focused on water pollution, accounted for 72% of the cumulative model weight and had the lowest AICc score, indicating its superior explanatory power. Nevertheless, because water pollution was identified as a mediator, Model 2 provides the best overall fit for describing the interplay between hemeroby, water pollution, and SR (Figure 6B).

The results for Shannon–Weiner plant species diversity (SD) indicate significant negative direct effects of hemeroby and water pollution in Models 1 and 3 (Model 1: B = −0.300, *p* = 0.000; Model 3: B = −0.583, *p* = 0.001). These findings suggest that increased human disturbance and reduced water quality independently decrease plant species diversity. However, in Model 2, where the combined effects of hemeroby and water pollution were assessed, neither variable showed a significant effect on plant species diversity (Figure 5). This indicates that water pollution does not mediate the relationship between hemeroby and species diversity. AIC model selection further supports this conclusion. Model 1, focusing on the direct effects of hemeroby, was identified as the best fit, accounting for 61% of the cumulative model weight and having the lowest AICc score. These results suggest that direct human disturbances affecting riparian habitats have a greater impact on species diversity than indirect effects mediated through water pollution (Figure 6A).

For species evenness, the results of Models 1 and 3 revealed negative but non‐significant direct effects of hemeroby and water pollution (Model 1: *B* = −0.091, *p* = 0.588; Model 3: *B* = −0.280, *p* = 0.119). However, in Model 2, which examined the combined effects of both variables, water pollution gained statistical significance (*B* = −0.803, *p* = 0.029). This suggests that water pollution mediates the relationship between hemeroby and species evenness. The AIC model selection also supports this conclusion, with Model 2 accounting for 49% of the cumulative model weight and having the lowest AICc score. These findings demonstrate that water pollution mediates the negative effects of hemeroby on species evenness, making Model 2 the best fit to describe this relationship (Figure 6C).

Generally, the results indicate that human disturbances have a direct negative effect on riparian plant species diversity, and water pollution plays a critical mediating role, amplifying the negative effects of human disturbances.

## 4 Discussion

The study results revealed that the rivers had moderately high plant SR. This aligns with Kark (2017), who reported that ecotones tend to harbor high biodiversity across spatial scales, including community‐level SR and intra‐species morphological or genetic diversity. Given that this study was conducted within the ecotone between aquatic and terrestrial ecosystems along the rivers, this transitional nature likely explains the observed moderate SR. Similarly, a study by Van Rensburg et al. (2009) demonstrated that SR is negatively correlated with increasing distance from transition zones, reinforcing the richness typically found in such areas.

The Fabaceae, Cyperaceae, Poaceae, Asteraceae, and Lamiaceae families dominated the study area. This may be attributed to a combination of traits possessed by these families, including efficient long‐distance dispersal, successful establishment, ecological flexibility, disturbance tolerance, and the ability to change ecosystems by modifying the dynamics of fire and mammalian herbivory (Linder et al. 2018). In the case of the Sal and Zuari rivers, the fact that the hydrological regimes change during the monsoon and pre‐monsoon seasons in Goa may also explain why these families are most common. Plants from these families likely possess adaptive mechanisms enabling them to tolerate environmental changes, such as periodic flooding and drought conditions. This ecological adaptability allows them to thrive along the dynamic riverbanks, further explaining their prevalence in this study.

Beta diversity varied along the rivers, with sites in close proximity being less dissimilar than those farther apart. This pattern is consistent with ecological distance–decay relationships, where spatially closer sites share more similar environmental conditions and biotic interactions, leading to greater compositional similarity (Legendre et al. 2005). Remarkably, sites at corresponding positions along the two rivers also exhibited less dissimilarity than those far apart. This suggests that shared environmental gradients may exert similar selective pressures on plant communities in analogous positions. For this case, the changes in water salinity may have contributed to this relationship since the salinity of both the Sal and Zuari rivers increased with river flow toward the Indian Ocean. Increased salinity caused halophytes, particularly mangroves and their associates, to begin to emerge by the third station along both rivers, enhancing the stations' positional similarity. These results support the notion that identical riverine areas can experience convergent ecological conditions due to hydrological and positional factors (Tonkin et al. 2018).

The study also identified significant effects of anthropogenic pollution on plant composition along the rivers. Sewage disposal and urbanization emerged as the most impactful activities, while fishing had the least influence. This gradient of impact aligns with previous research, which has shown that high nutrient inputs from sewage and urban runoff significantly alter aquatic and riparian vegetation by favoring nutrient‐tolerant species (Knutson et al. 2004). More still, grasses from the family Poaceae were notably associated with areas impacted by sewage disposal. This finding aligns with previous observations that nutrient enrichment promotes the growth of fast‐growing grasses adapted to high nitrogen levels (Dodds et al. 2008).

In contrast, herbs thrived in forested areas with minimal human disturbance. This reflects the importance of intact vegetation for maintaining biodiversity and habitat complexity. Herbs are easily destroyed due to trampling by human movements; hence, they will not survive easily in disturbed areas (Laurance et al. 2011). Urbanization is associated with the dominance of trees. Human‐modified environments, which select woody species tolerant of urban stressors such as pollution and compacted soils, may account for this. Alternatively, this could also be due to trees' ability to survive human trampling since they will be bypassed during movement, unlike shrubs and herbs. On the other hand, fishing activities positively correlated with mangroves and their associates. This was likely due to the proximity of mangroves to fishing zones along the ocean, given their status as halophytes and their role as nurseries for fish species, as noted by Alongi (2012).

The findings of this study demonstrate that anthropogenic disturbances (hemeroby) and water pollution have a significant negative impact on plant species diversity in riparian ecosystems. This aligns with previous research, such as Dosskey et al. (2010), which documented how human activities degrade water quality in rivers, leading to declines in plant diversity along their banks. Human activities, such as channelization, significantly alter river morphology, resulting in increased flow velocity and changes in flood regimes. These environmental shifts disrupt riparian plant habitats, reducing diversity as some species fail to adapt or lose their competitive advantage, resulting in decreased abundance (Wohl 2017; Nilsson et al. 2005). For instance, in this study, the fifth station exhibited notably low plant diversity, likely due to large patches of the riverbanks being reinforced with stone bricks and water being diverted into fish farms. This habitat modification creates unfavorable conditions for riparian vegetation, thereby limiting plant SR and diversity.

This study observed that urbanized areas, particularly along the River Sal, exhibited reduced vegetation cover. This may be likely due to high levels of runoff pollution and edge effects. The compounded effects of water flow alterations and soil texture changes further accelerate declines in riparian plant diversity. Arheimer and Lindström (2019) noted that urbanization intensifies these pressures by increasing the proportion of impermeable surfaces, reducing soil permeability, and altering groundwater dynamics. These changes elevate water flow velocity and heighten pollutant loads in water bodies (Grizzetti et al. 2017). River Sal's more extensive urban development compared to River Zuari likely explains its higher pollution levels and lower plant diversity.

Likewise, anthropogenic disturbances, including damming, channeling, and recreational activities, also contribute to reduced species diversity and composition by decreasing the number of open patches available for plant establishment (Kuglerová et al. 2017). Reduced interaction between the riparian zone and the river itself limits the dispersal of plant propagules in the stream, thereby affecting SR (Jansson et al. 2005). This may explain the observed differences in plant SR across study stations, even when environmental conditions, such as salinity, were similar. For instance, stations four and five showed considerable differences in plant richness despite both being in saline environments.

The results show that water pollution mediates the negative effects of anthropogenic disturbances on plant species diversity. This finding is consistent with studies that highlight water quality as a critical driver of biodiversity loss in riparian ecosystems (Grizzetti et al. 2017; Kuglerová et al. 2017). Increased human disturbances lead to pollution from urban runoff, agricultural inputs, and sedimentation, which degrades water quality and creates unfavorable conditions for plant growth. The mediation effect observed here suggests that water pollution is a primary mechanism through which human disturbances reduce SR and diversity, a pattern corroborated by Arheimer and Lindström (2019). Habitat alterations caused by human disturbances, such as land clearing, soil compaction, and altered hydrology, have both direct and indirect impacts on species diversity (Naiman et al. 2005). Human disturbances may reduce niche availability and structural complexity in riparian zones, directly diminishing species diversity. Nilsson et al. (2005) reported similar findings, emphasizing the role of physical habitat changes in shaping riparian plant communities.

Meanwhile, the results revealed that hemeroby and water pollution negatively affect species evenness, with water pollution having a mediating role. This indicates that water quality deterioration amplifies the impacts of human disturbances, leading to uneven species distributions. Pollutants, such as excess nutrients and heavy metals, disproportionately favor certain species while disadvantaging others, leading to lower evenness (Dodds et al. 2008). Additionally, the significant mediation effect highlights how water pollution exacerbates the impacts of hemeroby on riparian vegetation structures. Similar patterns have been observed by Grizzetti et al. (2017), who report that urban runoff and agricultural pollutants disrupt ecosystem balance, reducing species evenness.

These results underscore the need for targeted conservation measures that tackle habitat issues directly and indirectly through water pollution. The detrimental effects on plant species diversity could be considerably lessened by initiatives to improve water quality, such as improved riparian buffer restoration, sustainable agriculture methods, and improved sewage management. Additionally, maintaining species diversity in riparian zones requires minimizing direct human impacts, such as channel modification, urbanization, and land clearing.

## 5 Conclusion

The riparian plant species diversity along the Sal and Zuari rivers is moderate. This implies that the riparian vegetation has suffered significant human disturbances that have reduced its abundance along the rivers' ecotones. As the seasons changed from monsoon to pre‐monsoon, the riparian plants became less diverse, rich, and even. These traits also changed between study stations as human disturbance patterns and salinity levels changed.

The levels of anthropogenic pollution along the Sal and Zuari rivers were moderately high. The level of human disturbance along the rivers was alpha eu‐hemerobic, which is characterized by strong human impacts and the environment being far from natural. The main human activities along the rivers were sewage disposal, construction, urbanization, and fish farming. Therefore, we can conclude that both River Sal and River Zuari are under significant anthropogenic pressure, potentially leading to further increases in their pollution levels.

Anthropogenic pollution has a significant effect on plant composition; as such, the nature of human activity will determine the plant composition of that area. Anthropogenic disturbances (hemeroby) and water pollution negatively impact plant diversity in riparian ecosystems. In addition, water pollution plays a pivotal role in mediating the effects of human disturbances on plant diversity, thus serving as a significant pathway through which human disturbances impact plant species diversity. Effective conservation efforts should thus prioritize mitigating water pollution and reducing direct human disturbances to safeguard biodiversity and ecosystem functionality in riparian zones.

A thorough review of literature shows that the results of this study have added a new body of knowledge to the existing literature. For example, this study has demonstrated how hemeroby interacts with water pollution to affect plant species diversity. Most studies focus on hemeroby, neglecting its indirect role through water pollution. Also, there is no published study on the relationship between anthropogenic activities and plant species diversity along the Zuari or Sal rivers, despite their already highlighted high pollution levels and remediation initiatives, of which riparian vegetation can play a very significant role. This study bridged this gap.

## Conflicts of Interest

The authors declare no conflicts of interest.

## Supporting information


Data S1.

## Data Availability

The data that supports the findings of this study is provided in the Supporting Information.

## Appendix A

### List of Plant Families

**Table:**

Family	Species	Growth form
Acanthaceae	*Acanthus ilicifolius* L.	Shrub
	*Asystasia gangetica* (L.) T. Anderson	Herb
	*Avicennia marina* (Forssk.) Vierh.	Shrub
	*Avicennia officinalis* L.	Tree
Aizoaceae	*Sesuvium portulacastrum* (L.) L.	Herb
Amaranthaceae	*Alternanthera ficoidea* (L.) P. Beauv.	Herb
	*Alternanthera sessilis* (L.) R.Br. ex DC.	Herb
	*Amaranthus blitum* L.	Herb
Anacardiaceae	*Mangifera indica* L.	Tree
Arecaceae	*Cocos nucifera* L.	Tree
	*Caryota urens* L.	Tree
Araceae	*Amorphophallus paeoniifolius* (Dennst.) Nicolson	Herb
	*Colocasia esculenta* (L.) Schott.	Herb
Asteraceae	*Acmella radicans* (Jacq.) R.K. Jansen	Herb
	*Ageratum conyzoides* L.	Herb
	*Calyptocarpus vialis* Less.	Herb
	*Chromolaena odorata* (L.) R.M.King & H.Rob.	Shrub
	*Eclipta prostrata* (L.) L.	Herb
	*Sphagneticola trilobata* (L.) Pruski	Herb
	*Tridax procumbens* L.	Herb
Balsaminaceae	*Impatiens balsamina* L.	Herb
Bignoniaceae	*Dolichandrone spathacea* (L.f.) K.Schum.	Tree
Boraginaceae	*Heliotropium indicum* L.	Herb
Cannabaceae	*Trema orientalis* (L.) Bl.	Tree
Cleomaceae	*Cleome viscosa* L.	Herb
Commelinaceae	*Murdannia nudiflora* (L.) Bren.	Herb
	*Tradescantia fluminensis* L.	Herb
Convolvulaceae	*Ipomoea corymbosa* (L.)Roth ex Roem. & Schult.	Herb
	*Ipomoea pes‐caprae* (L.) R. Br.	Herb
	*Ipomoea violacea* L.	Herb
	*Merremia hederacea* (Burm.f.) Hallier f.	Herb
Costaceae	*Cheilocostus speciosus* (J.Koenig) C.D.Specht	Herb
Cucurbitaceae	*Cucumis anguria* L.	Herb
Cyperaceae	*Cyperus articulatus* L.	Herb
	*Cyperus compressus* L.	Herb
	*Cyperus difformis* L.	Herb
	*Cyperus exaltatus* Retz.	Herb
	*Cyperus iria* L.	Herb
	*Cyperus javanicus* Houtt.	Herb
	*Cyperus longus* L.	Herb
	*Cyperus rotundus* L.	Herb
	*Cyperus strigosus* L.	Herb
	*Fimbristylis dichotoma* (L.) Vahl	Herb
	*Rhynchospora corymbosa* (L.) Britton	Tree
	Schoenoplectus lateriflorus (J.F. Gmel.) Lye	Herb
	*Schoenoplectus lacustris* (L.) Palla	Herb
Dioscoreaceae	*Dioscorea alata* L.	Herb
	*Dioscorea bulbifera* L.	Herb
Euphorbiaceae	*Excoecaria agallocha* L.	Tree
	*Grahmii sps*.	Shrub
	*Macaranga peltata (Roxb.) Müll.Arg*.	Tree
Fabaceae	*Abrus precatorius L*.	Shrub
	*Acacia chundra (Rottler) Willd*.	Tree
	*Acacia auriculiformis A. Cunn. ex Benth*.	Tree
	*Alysicarpus vaginalis (L.) DC*.	Herb
	*Caesalpinia crista L*.	Shrub
	*Canavalia gladiata (Jacq.) DC*.	Herb
	*Centrosema virginianum (L.)Benth*	Shrub
	*Crotalaria pallida Aiton*	Shrub
	*Crotaloria verrucosa L*.	Shrub
	*Derris trifoliata Lour*.	Shrub
	*Desmodium tortuosum (Sw.) DC*.	Herb
	*Geissaspis cristata Wight & Arn*.	Herb
	*Laburnum anagyroides Medik*.	Shrub
	*Mimosa pudica L*.	Herb
	*Pongamia pinnata (L.) Pierre*	Tree
	*Vigna vexillata (L.) A. Rich*	Tree
Lamiaceae	*Leucas aspera* (Willd.) Linn.	Herb
	*Leucas lavandulifolia* Sm.	Herb
	*Rotheca serrata (L.)* Steane and Mabb.	Shrub
	*Clerodendrum inerme* (L.) Gaertn.	Shrub
	*Clerodendrum infortunatum* L.	Shrub
	*Premna serratifolia L*.	Tree
Lecythidaceae	*Barringtonia acutangula L*.	Tree
Loranthaceae	*Loranthus sps. Jacq*.	Herb
Lygodiaceae	*Lygodium japonicum (Thunb.) Sw*.	Herb
Lythraceae	*Lagerstroemia speciosa L*.	Shrub
	*Sonneratia alba J. Sm*.	Tree
Malvaceae	*Ceiba pentandra • (L.) Gaertn*.	Tree
	*Thespesia populnea (L.) Soland. Ex Correa*	Tree
	*Urena lobata L*.	Shrub
Moraceae	*Artocarpus heterophyllus Lam*.	Tree
	*Ficus heterophylla L.f*.	Tree
	*Ficus hispida L. f*.	Tree
	*Ficus recemosa L*.	Tree
Moringaceae	*Moringa oleifera Lam*.	Tree
Myrtaceae	*Syzygium caryophyllatum (L.) Alston*	Tree
Nephrolepidaceae	*Nephrolepis sps*.	Herb
Pandanaceae	*Pandanus tectorius Parkison ex DuRoi*	Shrub
Pedaliaceae	*Sesamum indicum L*.	Herb
Phyllanthaceae	*Phyllanthus reticulatus Poir*.	Shrub
Piperaceae	*Peperomia pellucida (L.) Kunth*	Herb
Poaceae	*Axonopus compressus (Sw.) P. Beauv*.	Herb
	*Brachiaria mutica (Forssk.) Stapf*	Herb
	*Chloris barbata Sw*.	Herb
	*Cynodon dactylon (L.) Pers*.	Herb
	*Digitaria ciliaris (Retz.) Koeler*	Herb
	*Echinochloa colona (L.)*	Herb
	*Ehrharta erecta Lam*.	Herb
	*Eragrostis tenella (A.Rich.) Hochst. ex steud*	Herb
	*Eragrostis viscosa (Retz.) Trin*.	Herb
	*Hymenachne amplexicaulis (Rudge) Nees*	Herb
	*Ochlandra travancorica Bedd*.	Herb
	*Pseudosasa japonica (Siebold & Zucc. ex Steud.) Makino ex Nakai*	Herb
	*Sacciolepsis striata (L.) Nash*	Herb
Polygonaceae	*Persicaria glabra (Willd.) Gomez de la Maza*	Herb
	*Persicaria maculosa S. F. Gray*	Herb
	*Aglaomorpha quercifolia (L.) Hovenkamp & S. Linds*	Herb
Pontederiaceae	*Pontederia crassipes Mart*.	Herb
Pteridaceae	*Acrostichum aureum L*.	Shrub
Rhamnaceae	*Ziziphus jujuba Mill*.	Tree
	*Ziziphus mauritania Lam*.	Herb
Rhizophoraceae	*Bruguiera gymnorrhiza (L.) Lam*.	Tree
	*Kandelia candel (L.) Druce*	Shrub
	*Rhizophora mucronata Lamk*.	Tree
Rubiaceae	*Ixora coccinea L*.	Shrub
	*Mussaenda glabrata (Hook. f.) Hutch. ex Gamble*	Shrub
Salicaceae	*Salix alba L*.	Tree
	*Salvinia minima*	Herb
Smilacaceae	*Smilax china L*.	Herb
Thelypteridaceae.	*Cyclosorus interruptus (Willd.) H. Itô*	Herb
Verbenaceae	*Lantana camara* L.	Shrub
	*Tectona grandis* L.f	Tree
Vitaceae	*Leea indica* (Burm.f.) Merr. Lam.	Shrub
	*Vitis aestivalis* Michx.	Tree
	*Cayratia trifolia* (L.) Domin	Shrub
